# Vascular complications in 305 severely ill patients with COVID-19: a cohort study

**DOI:** 10.1590/1516-3180.2022.0171.R2.17102022

**Published:** 2022-12-19

**Authors:** Rebeca Mangabeira Correia, Brena Costa Santos, Ana Alyra Garcia Carvalho, Libnah Leal Areias, Danielle Akemi Bergara Kuramoto, Mariana Raffo Pereda, Ana Laura e Silva Aidar, Caroline Nicacio Bessa Clezar, Marcello Erich Reicher, Jorge Eduardo de Amorim, Ronald Luiz Gomes Flumignan, Luis Carlos Uta Nakano

**Affiliations:** I MD. Master’s Student, Division of Vascular and Endovascular Surgery, Universidade Federal de São Paulo (UNIFESP), São Paulo (SP), São Paulo, Brazil.; II MD. Master’s Student, Division of Vascular and Endovascular Surgery, Universidade Federal de São Paulo (UNIFESP), São Paulo (SP), São Paulo, Brazil.; III MD. Master’s Student, Division of Vascular and Endovascular Surgery, Universidade Federal de São Paulo (UNIFESP), São Paulo (SP), São Paulo, Brazil.; IV MD. Master’s Student, Division of Vascular and Endovascular Surgery, Universidade Federal de São Paulo (UNIFESP), São Paulo (SP), São Paulo, Brazil.; V MD. Master’s Student, Division of Vascular and Endovascular Surgery, Universidade Federal de São Paulo (UNIFESP), São Paulo (SP), São Paulo, Brazil.; VI MD. Master’s Student, Division of Vascular and Endovascular Surgery, Universidade Federal de São Paulo (UNIFESP), São Paulo (SP), São Paulo, Brazil.; VII MD. Master’s Student, Division of Vascular and Endovascular Surgery, Universidade Federal de São Paulo (UNIFESP), São Paulo (SP), São Paulo, Brazil.; VIII MD. Doctoral Student, Division of Vascular and Endovascular Surgery, Universidade Federal de São Paulo (UNIFESP), São Paulo (SP), São Paulo, Brazil.; IX MD, PhD. Affiliate Professor, Division of Vascular and Endovascular Surgery, Universidade Federal de São Paulo (UNIFESP), São Paulo (SP), Sao Paulo, Brazil.; X MD, PhD. Adjunct Professor, Division of Vascular and Endovascular Surgery, Universidade Federal de São Paulo (UNIFESP), São Paulo (SP), São Paulo, Brazil.; XI MD, PhD. Full Professor, Division of Vascular and Endovascular Surgery, Universidade Federal de São Paulo (UNIFESP), São Paulo (SP), São Paulo, Brazil.; XII MD, PhD. Full Professor, Division of Vascular and Endovascular Surgery, Universidade Federal de São Paulo (UNIFESP), São Paulo (SP), São Paulo, Brazil.

**Keywords:** Mortality, COVID-19, Cohort studies, Peripheral vascular diseases, Critical care, Vascular complications, SARS-CoV-2 infection, Retrospective analysis, 30-days follow-up

## Abstract

**BACKGROUND:**

Although an association has been made between coronavirus disease 2019 (COVID-19) and microvascular disease, data on vascular complications (other than venous thromboembolism) are sparse.

**OBJECTIVE:**

To investigate the vascular complications in severely ill patients hospitalized with COVID-19 and their association with all-cause mortality.

**DESIGN AND SETTING:**

This cohort study was conducted at the Universidade Federal de São Paulo, Brazil.

**METHODS:**

All 305 consecutive patients diagnosed with COVID-19 and hospitalized in the intensive care unit (ICU) of a tertiary university hospital from April 2 to July 17, 2021, were included and followed up for 30 days.

**RESULTS:**

Of these, 193 (63.3%) were male, and the mean age was 59.9 years (standard deviation = 14.34). The mortality rate was 56.3% (172 patients), and 72 (23.6%) patients developed at least one vascular complication during the follow-up period. Vascular complications were more prevalent in the non-survivors (28.5%) than in the survivors (17.3%) group and included disseminated intravascular coagulation (DIC, 10.8%), deep vein thrombosis (8.2%), acrocyanosis (7.5%), and necrosis of the extremities (2%). DIC (adjusted odds ratio (aOR) 2.30, 95% confidence interval (CI) 1.01–5.24, P = 0.046) and acrocyanosis (aOR 5.21, 95% CI 1.48–18.27, P = 0.009) were significantly more prevalent in the non-survivors than in the survivors group.

**CONCLUSION:**

Vascular complications in critically ill COVID-19 patients are common (23.6%) and can be closely related to the mortality rate (56.3%) until 30 days after ICU admission. Macrovascular complications have direct implications for mortality, which is the main outcome of the management of COVID-19.

**REGISTRATION:**

RBR-4qjzh7 (https://ensaiosclinicos.gov.br/rg/RBR-4qjzh7).

## INTRODUCTION

Coronavirus disease 2019 (COVID-19), caused by severe acute respiratory syndrome coronavirus 2 (SARS-CoV-2), has rapidly grown into a pandemic and affected populations worldwide, challenging public health and healthcare systems.^1^ The clinical spectrum of COVID-19 comprises a wide range of clinical manifestations such as mild upper respiratory tract illness, severe pneumonia with respiratory failure, disseminated intravascular coagulation, and even death. At the beginning of the COVID-19 outbreak, the three primary symptoms of the disease were fever, cough, and dyspnea, as well as other less common symptoms, including muscle pain, anorexia, malaise, and headache. However, 2–10% of patients with COVID-19 present with gastrointestinal symptoms such as diarrhea, abdominal pain, and vomiting; therefore, fecal-oral transmission, other than via close contact through respiratory droplets, has been questioned.^2^

During the COVID-19 pandemic, the authors have explored the risk factors related to life-threatening conditions or mortality in severely ill people with COVID-19. Age > 70 years and male sex were first associated with worse prognosis in hospitalized patients with COVID-19 who underwent surgical procedures.^3^

Recently, some studies have discussed other complications of COVID-19, including acute coronary syndrome, arrhythmia, pulmonary hypertension, arterial and venous thrombosis, and coagulopathy, especially in critically ill patients.^4-11^ Some publications discuss vascular complications and the hypercoagulability status in severe COVID-19 disease, but the related management is still under discussion.^6,8,11^ Although some studies discuss cardiovascular complications mainly during the late phase of COVID-19, there are sparse and conflicting data regarding the vascular complications and the real burden and course of these complications.^12,13^

## OBJECTIVE

The aim of this study was to evaluate vascular complications in critically ill patients hospitalized with COVID-19 and investigate their association with all-cause mortality. Second, the association between baseline conditions and vascular complications, invasive mechanical ventilation, vasopressors, and mortality was studied.

## METHODS

### Ethical approval and registration

The local research ethics commission approved this study (3.966.152) on June 24, 2020, and the methods were prospectively registered (RBR-4qjzh7) at the Rebec portal and the International Clinical Trials Registry Platform (U1111-1252-1318), World Health Organization (WHO). The study was conducted in accordance with the Brazilian Ethical Review System for research involving human beings and conformed to the Declaration of Helsinki of the World Medical Association (June 1964) and subsequent amendments. This study was conducted and reported in accordance with the Strengthening the Reporting of Observational Studies in Epidemiology Statement guidelines for reporting observational studies.^14^

### Patients and study design

The researchers enrolled consecutive patients with confirmed COVID-19 who were admitted to the intensive care unit (ICU) of a tertiary hospital between April 2 and July 17, 2021. The clinical outcomes were monitored until August 30, 2021. All patients were included retrospectively and were diagnosed with COVID-19 by RNA detection, following the WHO interim guidance.^15^ Although the researchers planned to exclude patients younger than 18 years old and those who died within 24 hours of admission to the ICU, all 305 enrolled patients were included, and none fulfilled the criteria for exclusion. All patients underwent a clinical examination, laboratory tests, and blood gas analysis. Symptoms were evaluated before ICU admission, that is, at hospital admission. All other outcomes are reported at the longest possible time point. At least one additional objective test, such as chest radiography, computed tomography, or duplex ultrasound, was used to confirm the diagnosis. All patients were treated in the ICU, with electrocardiography, non-invasive pressure, and peripheral oxygen saturation continuous motorization, and received prophylactic anticoagulants if there were no contraindications. Thromboprophylaxis was done according to the American Society of Hematology.^16^ The use of low molecular weight heparin was preferred for all patients except those with severe renal impairment and, due to the lack of evidence, no patient received the treatment dose for prophylactic purposes.^8,9,11,16^

### Outcomes of interest

As primary outcomes, the researchers analyzed the following vascular complications: disseminated intravascular coagulation (DIC), deep vein thrombosis (DVT), acrocyanosis, acute arterial occlusion, rhabdomyolysis, and distal extremity necrosis until 30 days after admission, discharge from the ICU, or death. All diagnoses were made after a specialized physical examination and at least one additional objective laboratory or imaging test. Second, the association between baseline conditions and vascular complications, invasive mechanical ventilation (IMV) requirement, vasopressors, and mortality was studied. All outcomes were monitored until August 30, 2021. The researchers evaluated core outcomes as predefined by the Core Outcome Measures in Effectiveness Trials Initiative for people with COVID-19.^17^

### Data collection

Epidemiological, demographic, clinical presentation, laboratory, imaging, and clinical data were extracted from electronic medical records. Two physicians independently checked all imputed data to avoid bias during the data collection and analysis processes. Details of the treatment measures (respiratory support, kidney replacement, and anticoagulant therapy) were also analyzed. Laboratory tests were collected at ICU admission, after 7, 10, 14, and 30 days, and at death, following the ICU routine.

The date of disease onset was defined as the day on which the first symptom or sign was observed. DIC was defined according to the International Society on Thrombosis and Hemostasis (ISTH) in 2001.^18^ The duration from disease onset to hospital admission, acute respiratory distress syndrome, and ICU admission were recorded.

### Laboratory procedures

The method used for laboratory confirmation of SARS-CoV-2 infection was throat swab real-time reverse transcriptase polymerase chain reaction. Blood cell count, alanine transaminase, aspartate transaminase, renal function, coagulation profile, C-reactive protein, liver function, D-dimer, troponin, and arterial blood gases were also determined. All patients underwent chest radiography or computed tomography. When there was clinical suspicion of DVT, the patient underwent a full bilateral lower limb venous duplex ultrasound scan (11 MHz linear transducer, Logic P6, GE Healthcare, Milwaukee, Wisconsin, United States). In cases of acute arterial occlusion, an additional arterial duplex ultrasound scan of the affected lower limb was performed to confirm clinical suspicion.

### Statistical analysis

Categorical variables were described as frequency rates (number of events and %), and continuous variables were described as mean or median values, in addition to standard deviation (SD) or minimum and maximum ranges when appropriate. For dichotomous variables, researchers calculated the odds ratio (OR), adjusted odds ratio (aOR), and 95% confidence intervals (CIs) by comparing baseline characteristics and outcomes of interest or by comparing different groups of patients. Age > 70 years and sex were used as confounding parameters to calculate the adjusted values. Mean differences (MD) and 95% CIs were used for continuous data and were compared using the t-test. Categorical variables were compared using the Pearson chi-square or Wald chi-square independence test, and multivariate analysis using multinomial logistic nominal regression or Poisson regression. Statistical analysis was based on all cases with valid data for all the variables in the model. Poisson regression models would be used when it was not possible to proceed with analyses using multinomial logistic nominal regression model. However, at the end of our study and after corrections, the Poisson regression model was not used. For adjusted and unadjusted analyses, we used the statistical software IBM SPSS Statistics for Windows (version 20.0, 2011, IBM Corp. Released, Armonk, New York, United States) and Minitab (version 17.1.0, 2013, Minitab Inc., State College, Pennsylvania, United States). In all tests, P < 0.05 was defined as statistically significant. IBM SPSS Statistics for Windows (version 20.0, 2011, IBM Corp. Released, Armonk, New York, United States) was used for forest plot graph development.

## RESULTS

### General characteristics

All 305 included patients were treated in the ICU due to the development of organ dysfunction; 193 (63.3%) were male, and the mean age was 59.94 (SD = 14.34) years. The mean age was higher in non-survivors than in survivors (MD 9.32, 95% CI 6.23–12.42, P < 0.00001), but there was no sex-related difference. All patients had at least one previous medical condition, and there was a mean of three comorbidities per patient (mean 3.04 [SD = 1.70]). Comorbidities and addiction were less prevalent in the survivors than in the non-survivors (MD 0.97, 95% CI 0.60–1.34, P < 0.00001), and hypertension (69.8%), diabetes (40.7%), chronic kidney disease (34.8%), and smoking (27.5%) were the most common in all samples. However, when adjusted for age and sex, hypertension (aOR 1.78, 95% CI 1.06–2.96, P = 0.028) and chronic kidney disease (aOR 1.89, 95% CI 1.14–3.12, P = 0.013) were significantly more prevalent in the non-survivors than in the survivors group (Table 1). The most common symptoms were dyspnea (81.3%), cough (63.9%), and fever (50.8%). Although there were more symptoms in survivors than in non-survivors (MD -0.57, 95% CI -0.99 to -0.15, P = 0.008), only chest pain (aOR 0.39, 95% CI 0.16–0.93, P = 0.033) was significantly less incident in the non-survivors group (Table 1).

**Table 1.** Demographics and baseline characteristics

	General (n = 305)	Survivors (n = 133)	Non-survivors (n = 172)	Difference*			Adjusted difference*		
				MD† | OR	CI (95%)	P value	MD† | OR	CI (95%)	P value
**Age, mean years (SD)**	59.94 (14.34)	54.68 (13.69)	64.01 (13.53)	9.32†	(6.23; 12.42)	0.000‡	9.32^†^	(6.23; 12.42)	0.000^‡^
Female, n (%)	112 (36.7)	46 (34.6)	66 (38.4)	0.85	(0.53; 1.36)	0.496	0.79	(0.49; 1.28)	0.34
Male, n (%)	193 (63.3)	87 (65.4)	106 (61.6)						
**Comorbidities**									
Mean per Patient, mean (SD)	3.04 (1.70)	2.50 (1.57)	3.46 (1.68)	0.97†	(0.60; 1.34)	0.000‡	0.97^†^	(0.60; 1.34)	0.000^‡^
Hypertension, n (%)	213 (69.8)	81 (60.9)	132 (76.7)	2.12	(1.29; 3.48)	0.003‡	1.78	(1.06; 2.96)	0.028^‡^
Diabetes, n (%)	124 (40.7)	48 (36.1)	76 (44.2)	1.40	(0.88; 2.23)	0.154	1.43	(0.88; 2.30)	0.145
Chronic kidney disease, n (%)	106 (34.8)	37 (27.8)	69 (40.1)	1.74	(1.07; 2.83)	0.026‡	1.89	(1.14; 3.12)	0.013^‡^
Current smoking, n (%)	84 (27.5)	28 (21.1)	56 (32.6)	1.81	(1.07; 3.06)	0.027‡	1.71	(0.99; 2.95)	0.056
**Symptoms**									
Mean per Patient, mean (SD)	4.02 (1.85)	4.35 (1.90)	3.78 (1.78)	-0,57†	(-0,99; -0,15)	0.008‡	-0.57^†^	(-0.99; -0.15)	0.008^‡^
Dyspnea, n (%)	248 (81.3)	110 (82.7)	138 (80.2)	0.54	(0.30; 0.97)	0.039‡	0.92	(0.50; 1.68)	0.787
Dry cough, n (%)	195 (63.9)	92 (69.2)	103 (59.9)	0.53	(0.33; 0.86)	0.009‡	0.69	(0.42; 1.13)	0.145
Fever, n (%)	155 (50.8)	77 (57.9)	78 (45.3)	0.59	(0.37; 0.93)	0.023‡	0.71	(0.44; 1.15)	0.162
Asthenia, n (%)	118 (38.7)	47 (35.3)	71 (41.3)	0.56	(0.35; 0.90)	0.016‡	1.27	(0.78; 2.06)	0.332
Myalgia, n (%)	81 (26.6)	41 (30.8)	40 (23.3)	0.71	(0.43; 1.19)	0.197	0.72	(0.43; 1.23)	0.229
Hyporexia, n (%)	55 (18.0)	26 (19.5)	29 (16.9)	0.67	(0.37; 1.20)	0.182	0.7	(0.38; 1.29)	0.251
Vomiting, n (%)	40 (13.1)	20 (15.0)	20 (11.6)	0.74	(0.38; 1.45)	0.383	0.79	(0.40; 1.56)	0.497
Headache, n (%)	39 (12.8)	22 (16.5)	17 (9.9)	0.87	(0.45; 1.71)	0.696	0.58	(0.29; 1.17)	0.126
Anosmia, n (%)	37 (12.1)	20 (15.0)	17 (9.9)	0.86	(0.43; 1.71)	0.663	0.64	(0.32; 1.31)	0.223
Abdominal pain, n (%)	31 (10.2)	13 (9.8)	18 (10.5)	0.78	(0.38; 1.59)	0.493	0.92	(0.42; 2.02)	0.835
Chest pain, n (%)	26 (8.5)	17 (12.8)	9 (5.2)	1.34	(0.58; 3.12)	0.491	0.39	(0.16; 0.93)	0.033^‡^
Sweating, n (%)	5 (1.6)	3 (2.3)	2 (1.2)	1.15	(0.19; 6.97)	0.882	0.63	(0.10; 3.87)	0.619

CI = confidence interval; MD = mean difference; n = number of patients; OR = odds ratio; SD = standard deviation. *Comparison between surviving and non-surviving patients, ^†^MD, ^‡^P < 0.05 (t-test for non-adjusted and nominal regression multinomial logistics for adjusted (age > 70 years and sex) comparisons).

### Clinical manifestations

Of the 305 included patients, 172 (56.3%) died by the end of the follow-up on August 30, 2021. The mean hospitalization time of the included patients was 24.68 (SD = 20.98) days, and the mean time in the ICU was 15.14 (SD = 15.88) days. The non-survivors group stayed more time in the ICU (MD 6.41, 95% CI 3.03–9.79, P < 0.0001) and had less time until the first outcome of interest for this study (MD -4.43, 95% CI -8.45 to -0.41, P = 0.032) (Table 2).

**Table 2.** Clinical outcomes

Time mean (days)	General (n = 305)		Survivors (n = 133)		Non-survivors (n = 172)		Difference*			Adjusted difference*		
	Mean	SD	Mean	SD	Mean	SD	MD	CI (95%)	P value	MD	CI (95%)	P value
ICU	15.14	15.88	11.53	12.16	17.94	17.78	6.41	(3.03; 9.79)	0.000^†^	N/A	N/A	N/A
Hospitalization	24.68	20.98	25.65	18.66	24.05	22.62	-1.60	(-6.26; 3.06)	0.500	N/A	N/A	N/A
ICU time until the outcome	20.01	17.86	22.50	17.48	18.08	17.96	-4.43	(-8.45; -0.41)	0.032^†^	N/A	N/A	N/A
Symptom onset to mechanical ventilation	8.96	6.68	8.32	4.85	9.24	7.33	0.92	(-0.98; 2.82)	0.342	N/A	N/A	N/A
**Outcomes**	**General (n = 305)**		**Survivors (n = 133)**		**Non-Survivors (n = 172)**		Difference*			Adjusted Difference*		
	**n**	**%**	**n**	**%**	**n**	**%**	**OR**	**CI (95%)**	**P value**	**OR**	**CI (95%)**	**P value**
Disseminated intravascular coagulation, n (%)	33	10.8	11	8.2	24	13.9	2.23	(1.00; 4.98)	0.050^†^	2.30	(1.01; 5.24)	0.046^†^
Deep vein thrombosis, n (%)	25	8.2	9	6.8	14	11.6	0.98	(0.43; 2.24)	0.967	0.87	(0.37; 2.04)	0.742
Acrocyanosis, n (%)	23	7.5	3	2.2	20	8.1	5.70	(1.66; 19.62)	0.005^†^	5.21	(1.48; 18.27)	0.009^†^
Necrosis of extremities, n (%)	6	2.0	2	1.5	5	2.9	3.95	(0.46; 34.24)	0.212	4.76	(0.54; 42.17)	0.161
Acute arterial occlusion, n (%)	4	1.3	1	0.7	2	1.1	0.77	(0.11; 5.54)	0.796	0.98	(0.14; 7.12)	0.985
Rhabdomyolysis, n (%)	1	0.3	0	0	1	0.6	2.34	(0.09; 57.79)	0.604	N/A	N/A	N/A
Complication free, n (%)	233	76.4	110	82.7	123	71.5	N/A	N/A	N/A	N/A	N/A	N/A
IMV requirement, n (%)	180 (59.0)		52 (39.1)		128 (74.4)		4.53	(2.78; 7.38)	0.000^†^	5.07	(3.03; 8.50)	0.000^†^
Vasopressors, n (%)	168 (55.1)		75 (56.4)		93 (54.1)		0.91	(0.58; 1.44)	0.686	0.856	(0.54; 1.37)	0.517

CI = confidence interval; IMV = invasive mechanical ventilation; MD = mean difference; n = number of patients; OR = odds ratio; SD = standard deviation. ^*^Comparison between surviving and non-surviving patients; ^†^P < 0.05 (t-test for non-adjusted and nominal regression multinomial logistics for adjusted (age > 70 years and sex) comparisons).

Seventy-two (23.6%) patients developed at least one vascular complication during the follow-up period. Among all the vascular complications identified, DIC (10.8%), DVT (8.2%), acrocyanosis (7.5%), and necrosis of the extremities (2%) were the most common. DIC (aOR 2.30, 95% CI 1.01–5.24, P = 0.046) and acrocyanosis (OR 5.21, 95% CI, 1.48–18.27; P = 0.009) were significantly more common in the non-survivors than in the survivors group (Table 2). DVT, necrosis of the extremities, acute arterial occlusion, and rhabdomyolysis were also more common in the non-survivor group, but the difference was not significant (Table 2).

Endovenous vasopressor medicines and IMV were required in 55.1% and 59% of the patients, respectively. Non-survivors had significantly higher IMV requirements than survivors (aOR 5.07, 95% CI 3.03–8.50, P < 0.0001) (Table 2).

**Figure 1.**
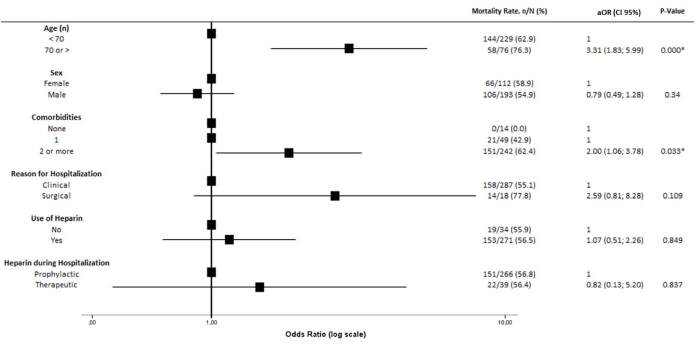
Association between baseline characteristics and all-cause mortality.

**Figure 2.**
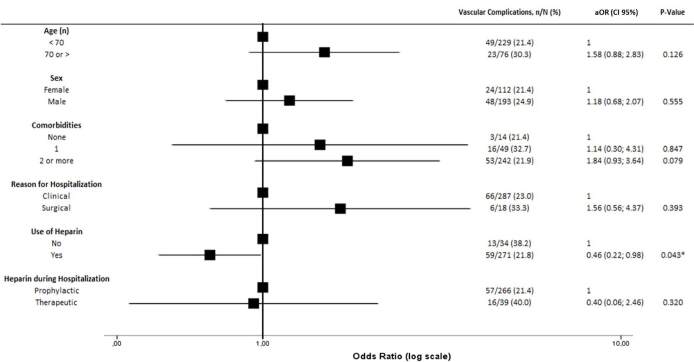
Association between baseline characteristics and vascular complications.

**Figure 3.**
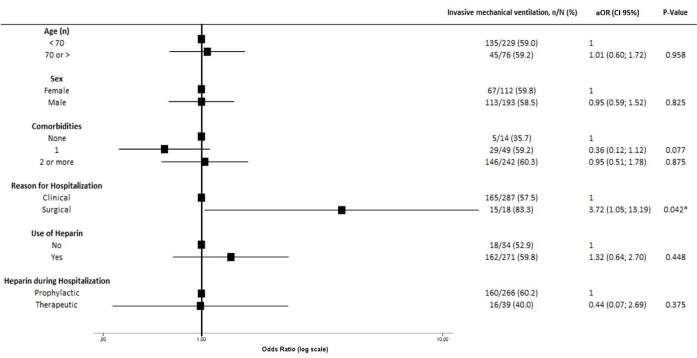
Association between baseline characteristics and invasive mechanical ventilation.

**Figure 4.**
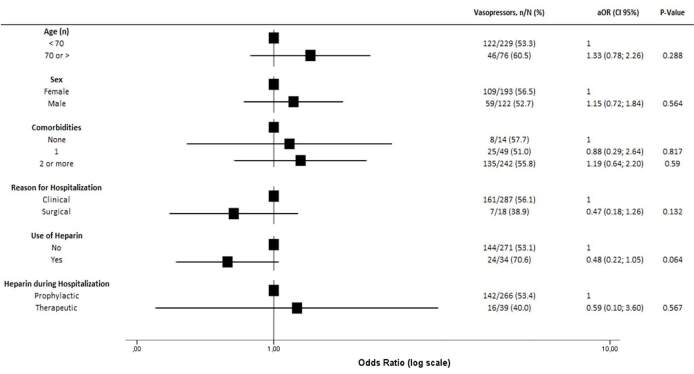
Association between baseline characteristics and vasopressor necessity.

Investigating the association between baseline characteristics and clinically relevant outcomes, there was more death in patients older than 70 years (aOR 3.31, 95% CI 1.83–5.99, P < 0.0001) and in those who presented with two or more comorbidities (aOR 2.00, 95% CI 1.06–3.78, P = 0.033) (Figure 1). Among all assessed baseline risk factors, only the previous use of heparin was associated with a decreased incidence of vascular complications (aOR 0.46, 95% CI 0.22–0.98; P = 0.043) (Figure 2). There was a greater need for IMV in patients who were hospitalized for surgical reasons (aOR 3.72, 95% CI 1.05–13.19, P = 0.042) (Figure 3). None of the baseline risk factors evaluated (age, sex, comorbidities, reason for hospitalization, use of heparin, and heparin dose used during hospitalization) were associated with any difference in the necessity of vasopressor agents during hospitalization (Figure 4).

The researchers assumed an OR of 1 for control baseline characteristics.

aOR = adjusted odds ratio; N = total number of patients; n = number of events.

^*^P < 0.05 (nominal regression multinomial logistics; IBM SPSS Statistics for Windows).

The researchers assumed an OR of 1 for control baseline characteristics.

aOR = adjusted odds ratio; N = total number of patients; n = number of events.

^*^P < 0.05 (nominal regression multinomial logistics; IBM SPSS Statistics for Windows); ^†^disseminated intravascular coagulation, deep vein thrombosis, acrocyanosis, necrosis of extremities, acute arterial occlusion, and rhabdomyolysis.

The researchers assumed an OR of 1 for control baseline characteristics.

aOR = adjusted odds ratio; N = total number of patients; n = number of events.

^*^P < 0.05 (nominal regression multinomial logistics; IBM SPSS Statistics for Windows).

The researchers assumed an OR of 1 for control baseline characteristics.

aOR = adjusted odds ratio; N = total number of patients; n = number of events.

^*^P < 0.05 (nominal regression multinomial logistics; IBM SPSS Statistics for Windows).

### Laboratory findings

**Figure 5.**
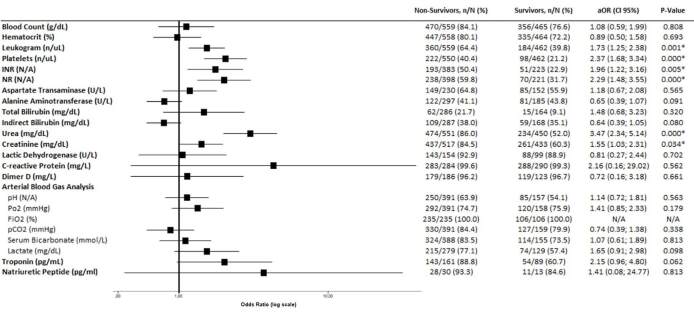
Laboratory findings and mortality prediction.

**Figure 6.**
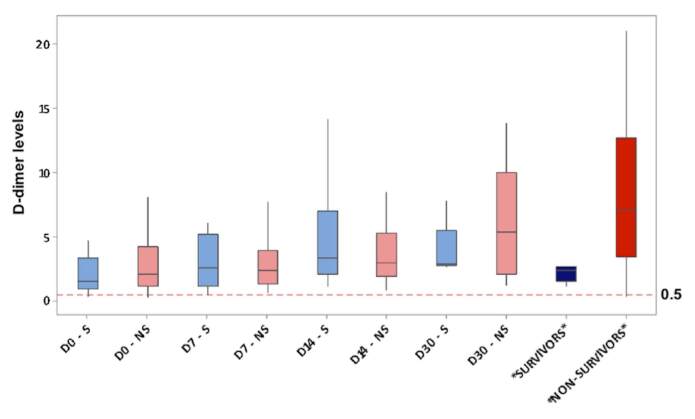
D-dimer levels^*^ of all patients.

Laboratory tests were performed on all patients. See Appendix 1 for the full local reference range used and Appendix 2 for the full laboratory testing results. Tests with values outside the local reference range were considered an event of interest, and the results were compared between non-surviving and surviving patients (Figure 5). Non-surviving patients presented significantly abnormal values for leukogram, platelets, international normalized ratio, normalized ratio, urea, and creatinine compared with surviving patients in the adjusted analysis (Appendix 2 and Figure 5). Notably, the baseline D-dimer count was significantly higher in all 305 patients during the study period (Figure 6). According to the ISTH diagnostic criteria for DIC, 33 patients (10.8%) matched the grade of overt DIC (≥ 5 points). The criteria were matched in the later stages of COVID-19. In our enrolled patients with DIC, all had a high D-dimer count, that is, more than five times the upper normal limit.

FiO2 = fraction of inspired oxygen; INR = international normalized ratio; N = total number of patients; n = number of events; NR = normalized ratio; aOR, adjusted odds ratio; pCO2, partial pressure of carbon dioxide; pO2, partial pressure of oxygen.

*P < 0.05 (t-test).

D0 = date of admission; D7 = day 7; D14 = day 14; D30 = day 30; NS = non-survivors; S = survivors.

^*^Values in mg/L.

## DISCUSSION

Patients with confirmed COVID-19 are commonly prone to in-hospital mortality and an elevated rate of thromboembolic events, including other vascular complications.^8,9,23^ A higher D-dimer level was observed in all patients admitted to the ICU, which suggests an association with the severity of the disease.^5^ The incidence of vascular complications were DIC = 10.8%, 8.2%, 7.5%, 2%, 1.3%, and 0.3% up to one month. The risk factors associated with death were age > 70 years and the presence of two or more comorbidities, while the risk factors associated with the necessity of invasive mechanical ventilation were hospitalization for surgical reasons in critically ill patients with COVID-19.

All 305 consecutive severely ill patients with confirmed COVID-19 were followed up for at least 30 days or until death. The majority were male (n = 193, 63.3%) and older adults (mean age 59.94 years [SD = 14.34]). Similarly, SARS-CoV-2 has been reported to infect more males than females.^19^

Of the 305 patients, 142 (79 %) had more than two comorbidities and chronic underlying diseases, including hypertension (69.8%), diabetes (40.7%), chronic kidney disease (34.8%), and smoking (27.5%). Previous studies have indicated hypertension and diabetes as highly prevalent in hospitalized patients with COVID-19, but we introduced chronic kidney disease as another highly incident comorbidity on this site.^20^ Our data also confirm the probable association with severe COVID-19 related outcomes such as chronic kidney disease.^20^

Of the 305 included patients, 172 (56.3%) died, and 72 (23.6%) developed at least one vascular complication (DIC, 10.8%; DVT, 8.2%; acrocyanosis, 7.5%; necrosis of extremities, 2%; acute arterial occlusion, 1.3%; and rhabdomyolysis, 0.3%) during the follow-up period. One patient (0.3 %) presented rhabdomyolysis, which is not a frequent complication related to novel coronavirus infection, but has already been described in the literature.^21^ Another study reported rates of 6.6% pulmonary embolism and 11.6% other cardiac complications in hospitalized patients with COVID-19.^22^ We reported other clinically relevant and highly prevalent vascular complications such as DIC, acrocyanosis, necrosis of extremities, acute arterial occlusion, and rhabdomyolysis. Our symptomatic DVT rate (8.2%) was similar to previous venous thromboembolism (VTE) rates of 11.2% in hospitalized patients, but was less than the rates (31% to 49%) reported for ICU patients.^8,9,23^

Thromboembolic events have been frequently described in patients with COVID-19, but the actual incidence of these events might be underestimated due to underdiagnosis and the low number of imaging tests performed.^24,25^ Further studies are necessary to determine the real incidence of these events and to improve the prevention and facilitate the diagnosis and treatment of thromboembolic complications in patients with COVID-19. As there is high certainty evidence against the use of a therapeutic dose of anticoagulation for prophylactic purposes in patients hospitalized with COVID-19, strategies for early VTE diagnosis, mainly in critically ill patients, could be helpful.^8,9,26^

Tang et al. reported a higher D-dimer level and a longer prothrombin time in non-surviving patients when compared to survivors, and a high rate of DIC manifested in the majority of deaths.^5^ It is well established that sepsis is one of the most common causes of DIC and that viral infection may develop into sepsis associated with organ dysfunction.^3^ However, in our analysis, D-dimer levels were elevated in all patients (mean 4.54, SD = 5.41 mg/L), that is, almost five times the local upper normal limit. Recent studies have associated D-dimer levels with a poor prognosis in people with COVID-19^5^; therefore, it is considered a typical marker related to hypercoagulability and thrombotic events, and it also has the potential to be used as an indicator for prognosis and progression of the disease.^27^ We observed DIC in 33 (10.8%) of all included patients and in 24 (15%) of the non-survivors group, differing from the currently available data, which reached 70% DIC occurrence in some samples.^5^ It can be explained by the fact that D-dimer levels were not measured daily in the ICU, only on admission. Hence, DIC was diagnosed based on the ISTH criteria in our sample.

The most prevalent symptoms at the onset of the illness were dyspnea (81.3%), cough (63.9%), and fever (50.8%), similar to those reported in other studies.^20^ However, other symptoms such as thoracic and abdominal pain, vomiting, and sweating were present in our patients, showing that coronavirus infection can present itself with a wide range of clinical manifestations.

Most patients required IMV (59%) and endovenous vasopressor medication (55.1%) in the ICU. All non-survivors developed acute respiratory failure, underwent oral intubation, and required vasopressor administration. Half of these patients needed hemodialysis as a result of acute kidney injury diagnosed using the Kidney Disease Improving Global Outcomes (KDIGO) clinical practice guidelines.^28^ Cheng et al. found a higher in-hospital death rate for patients with kidney abnormalities, showing that acute kidney injury or even chronic renal disease can contribute as a risk factor for a poor prognosis in COVID-19 patients.^29^

Benson et al.^30^ emphasized a high mortality rate of 11% after vascular and endovascular procedures (elective or urgent) during the pandemic period, even at lower rates (4%) of confirmed COVID-19 cases. COVIDSurg Collaborative et al.^31^ found that 30-day mortality in patients with COVID-19 increased from 7.4% to 40.8% in those with VTE, and Kollias et al.^32^ reported that severely ill patients with COVID-19 had high rates of pulmonary embolism (32%) and DVT (27%) despite prophylactic anticoagulation. In our study, the death rate was even higher: 55.1% in those hospitalized for clinical reasons and 77.8% in those hospitalized for surgical reasons. Most deaths also occurred during the first two weeks after admission to the ICU, which demonstrates that the length of stay in the ICU can be long, demanding more resources and meaning a longer time of intubation for these patients.

Our study had some limitations. First, this was a retrospective observational study based on the analysis of medical records. Although laboratory tests were performed for all patients, not all laboratory tests, including D-dimer and fibrinogen, were performed. Hence, their importance in the poor outcomes of these patients could be underestimated. However, establishing the incidence of highly relevant vascular complications is essential for providing better treatment for all patients with COVID-19.

Even more than two years after its outbreak, the COVID-19 pandemic is a global health issue. However, non-transmissible circulatory diseases remain the leading cause of disease burden worldwide.^33^ The high prevalence of related risk factors, such as hypertension (69.8%), diabetes (40.7%) and smoking (27.5%), reaffirmed their burden. Additionally, severely ill patients with COVID-19 are often followed by cardio-, cerebral-, and peripheral vascular complications, and clinicians prescribe pharmacological and non-pharmacological interventions to avoid complications such as VTE, acute limb ischemia, amputation, and death.^8,12,22,26^ However, there is no consensus regarding the impact of vascular complications on managing severely ill patients with COVID-19. The high prevalence of vascular complications (23.6%) in our study suggests that this impact may also be observed in severely ill patients with COVID-19.

## CONCLUSIONS

The high death rate (56.3%) and the relatively high incidence of all vascular complications (23.6%) demonstrate the need to improve specific diagnostic and prevention strategies to manage COVID-19 complications.

## Appendix 1. Local reference range for laboratory tests

**Table:**

Laboratory tests	Reference range
Blood count (g/dL)	13.5–17.5
Hematocrit (%)	39–50
Leukogram (n/μL)	3,500–10,500
Platelets (n/μL)	150,000–450,000
INR (N/A)	0.8–1.2
NR (N/A)	0.8–1.2
Aspartate transaminase (U/L)	Up to 40
Alanine aminotransferase (U/L)	Up to 41
Total bilirubin (mg/dL)	Up to 1.0
Indirect bilirubin (mg/dL)	0.1–0.6
Urea (mg/dL)	10–50
Creatinine (mg/dL)	0.7–1.2
Lactic dehydrogenase (U/L)	Up to 250
C-reactive protein (mg/L)	Up to 1.0
D-dimer (mg/L)	Up to 0.5
Arterial blood gas analysis	
pH (N/A)	7.35–7.45
pO_2_ (mmHg)	80–100
FiO_2_ (%)	95–98
pCO_2_ (mmHg)	35–45
Serum bicarbonate (mmol/L)	22–26
Lactate (mg/dL)	4.5–14.4
Troponin (pg/mL)	Up to 14
Natriuretic peptide (pg/mL)	125–400

INR = international normalized ratio; NR = normalized ratio; U/L = units/liter; pH = potential of hydrogen; N/A = not available; pO_2_ = partial pressure of oxygen; FiO_2 =_ fraction of inspired oxygen; pCO_2 =_ partial pressure of carbon dioxide.

## Appendix 2. Laboratory testing

**Table:**

Laboratory testing	General (N)	Non-survivors	Survivors	Difference*			Adjusted difference*		
		n (%)	n (%)	OR	CI (95%)	P value	OR	CI (95%)	P value
**Blood Count (g/dL)**	1024	470 (84.1)	356 (76.6)	1.62	(1.18; 2.21)	0.002^†^	1.08	(0.59; 1.99)	0.808
**Hematocrit (%)**	1022	447 (80.1)	335 (72.2)	1.55	(1.16; 2.07)	0.003^†^	0.89	(0.5; 1.58)	0.693
**Leukogram (n/uL)**	1021	360 (64.4)	184 (39.8)	2.73	(2.12; 3.52)	0.000^†^	1.73	(1.25; 2.38)	0.001^†^
**Platelets (n/uL)**	1012	222 (40.4)	98 (21.2)	2.51	(1.90; 3.33)	0.000^†^	2.37	(1.68; 3.34)	0.000^†^
**INR (N/A)**	606	193 (50.4)	51 (22.9)	3.43	(2.36; 4.97)	0.000^†^	1.96	(1.22; 3.16)	0.005^†^
**NR (N/A)**	619	238 (59.8)	70 (31.7)	3.21	(2.27; 4.54)	0.000^†^	2.29	(1.48; 3.55)	0.000^†^
**Aspartate Transaminase (U/L)**	382	149 (64.8)	85 (55.9)	1.45	(0.95; 2.20)	0.082	1.18	(0.67; 2.08)	0.565
**Alanine Aminotransferase (U/L)**	482	122 (41.1)	81 (43.8)	0.89	(0.62; 1.30)	0.558	0.65	(0.39; 1.07)	0.091
**Total Bilirubin (mg/dL)**	450	62 (21.7)	15 (9.1)	2.75	(1.51; 5.01)	0.001^†^	1.48	(0.68; 3.23)	0.320
**Indirect Bilirubin (mg/dL)**	455	109 (38.0)	59 (35.1)	1.13	(0.76; 1.68)	0.542	0.64	(0.39; 1.05)	0.080
**Urea (mg/dL)**	1001	474 (86.0)	234 (52.0)	5.68	(4.19; 7.70)	0.000^†^	3.47	(2.34; 5.14)	0.000^†^
**Creatinine (mg/dL)**	950	437 (84.5)	261 (60.3)	3.60	(2.65; 4.89)	0.000^†^	1.55	(1.03; 2.31)	0.034^†^
**Lactic Dehydrogenase (U/L)**	253	143 (92.9)	88 (88.9)	1.62	(0.68; 3.91)	0.278	0.81	(0.27; 2.44)	0.702
**C-reactive Protein (mg/L)**	574	283 (99.6)	288 (99.3)	1.96	(0.18; 21.79)	0.582	2.16	(0.16; 29.02)	0.562
**Dimer D (mg/L)**	309	179 (96.2)	119 (96.7)	0.86	(0.25; 3.00)	0.812	0.72	(0.16; 3.18)	0.661
**Arterial Blood Gas Analysis**									
**pH (N/A)**	548	250 (63.9)	85 (54.1)	1.50	(1.03; 2.19)	0.034^†^	1.14	(0.72; 1.81)	0.563
**pO**_**2**_ **(mmHg)**	549	292 (74.7)	120 (75.9)	0.93	(0.61; 1.44)	0.756	1.41	(0.85; 2.33)	0.179
**FiO**_**2**_ **(%)**	341	235 (100.0)	106 (100.0)	2.21	(0.04; 112.19)	0.692	N/A	N/A	N/A
**pCO**_**2**_ **(mmHg)**	550	330 (84.4)	127 (79.9)	1.36	(0.85; 2.19)	0.200	0.74	(0.39; 1.38)	0.338
**Serum Bicarbonate (mmol/L)**	543	324 (83.5)	114 (73.5)	1.82	(1.16; 2.84)	0.008^†^	1.07	(0.61; 1.89)	0.813
**Lactate (mg/dL)**	408	215 (77.1)	74 (57.4)	2.50	(1.60; 3.90)	0.000^†^	1.65	(0.91; 2.98)	0.098
**Troponin (pg/mL)**	250	143 (88.8)	54 (60.7)	5.15	(2.69; 9.85)	0.000^†^	2.15	(0.96; 4.8)	0.062
**Natriuretic Peptide (pg/ml)**	43	28 (93.3)	11 (84.6)	2.54	(0.32; 20.38)	0.379	1.41	(0.08; 24.77)	0.813

CI = confidence interval; MD = mean difference; n = number of patients or tests; SD = standard deviation; U/L = units/liter; pH = potential of hydrogen; pO_2_ = partial pressure of oxygen; FiO_2 =_ fraction of inspired oxygen; pCO_2 =_ partial pressure of carbon dioxide. *Comparison between surviving and non-surviving patients. †P < 0.05 (t-test).
